# Supplementary material for: Different NIPBL requirements of cohesin-STAG1 and cohesin-STAG2
Source: Nat Commun. 2023 Mar 10;14:1326. doi: 10.1038/s41467-023-36900-7 (PMC10006224; doi:10.1038/s41467-023-36900-7)
Supplement: Supplementary file 1 — Supplementary Information [file 41467_2023_36900_MOESM1_ESM.pdf]

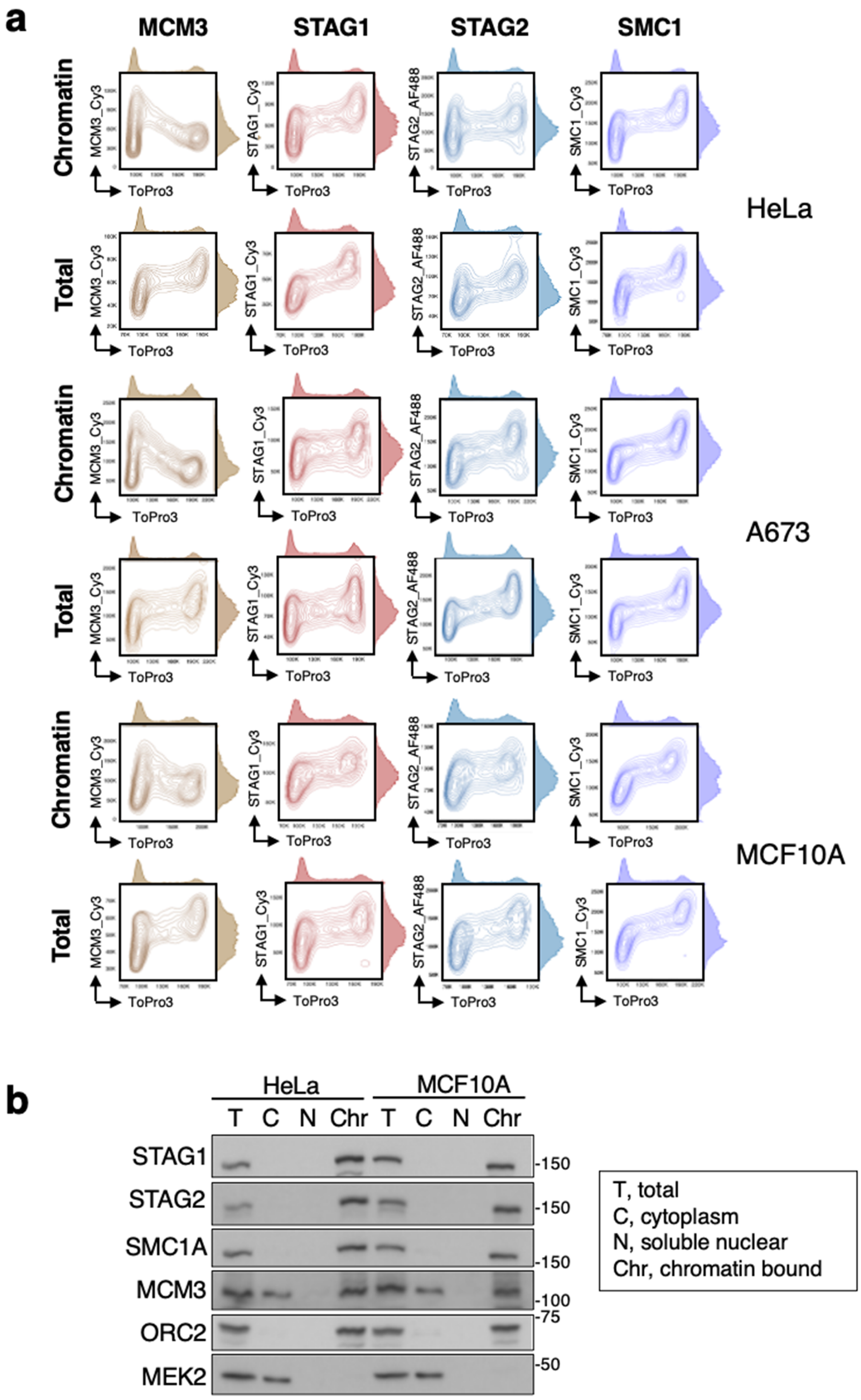

Supplementary Fig. 1 Chromatin association of the two cohesin variants by flow cytometry. **a** Flow cytometry analysis of asynchronously growing HeLa, Ewing sarcoma A673 and mammary epithelial MCF10A cells with the indicated antibodies. Results are shown as contour plots. Cells were either pre-extracted with detergent before fixation to measure chromatin-bound protein levels (Chromatin) or permeabilized after fixation to assess total levels in the cell (Total). For each map, the cell cycle profile according to DNA content appears on top while the distribution of antibody intensities is plotted on the right. **b** Immunoblot analysis of the indicated cellular fractions from HeLa and MCF10A cells. ORC2, a chromatin bound protein, and MEK2, a cytoplasmic kinase, were used as controls for the fractionation procedure. This is a single experiment.

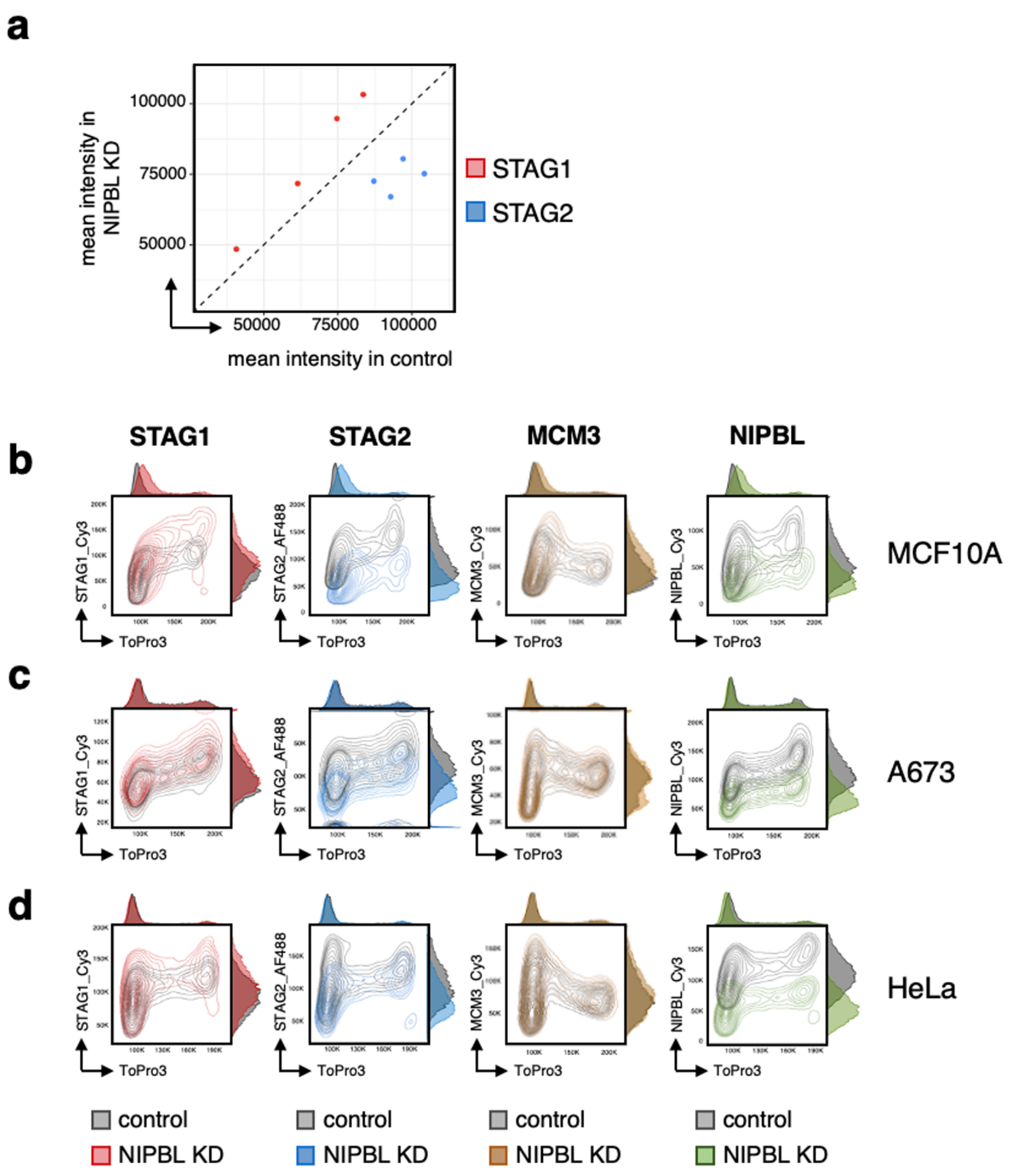

Supplementary Fig. 2 NIPBL KD affects cohesin-STAG1 and cohesin-STAG2 in opposite ways also in MCF10A and A673 cells. a Mean intensity values for STAG1 and STAG2 in control and NIPBL KD HeLa cells (n=4 experiments). The plot shows an increase in STAG1 signal and a decrease in STAG2 signal upon NIPBL KD, both statistically significant (p-values: 0.02 and 0.008686, respectively, using paired two-sided Student t Test). **b-d** Flow cytometry contour plots for chromatin-bound levels of the indicated proteins in control (grey plots) and NIPBL KD (colored plots) in MCF10A cells (b), A673 cells (c) and HeLa cells (d). In the latter experiment, a mixture of 4 siRNAs (smart pool) was used.

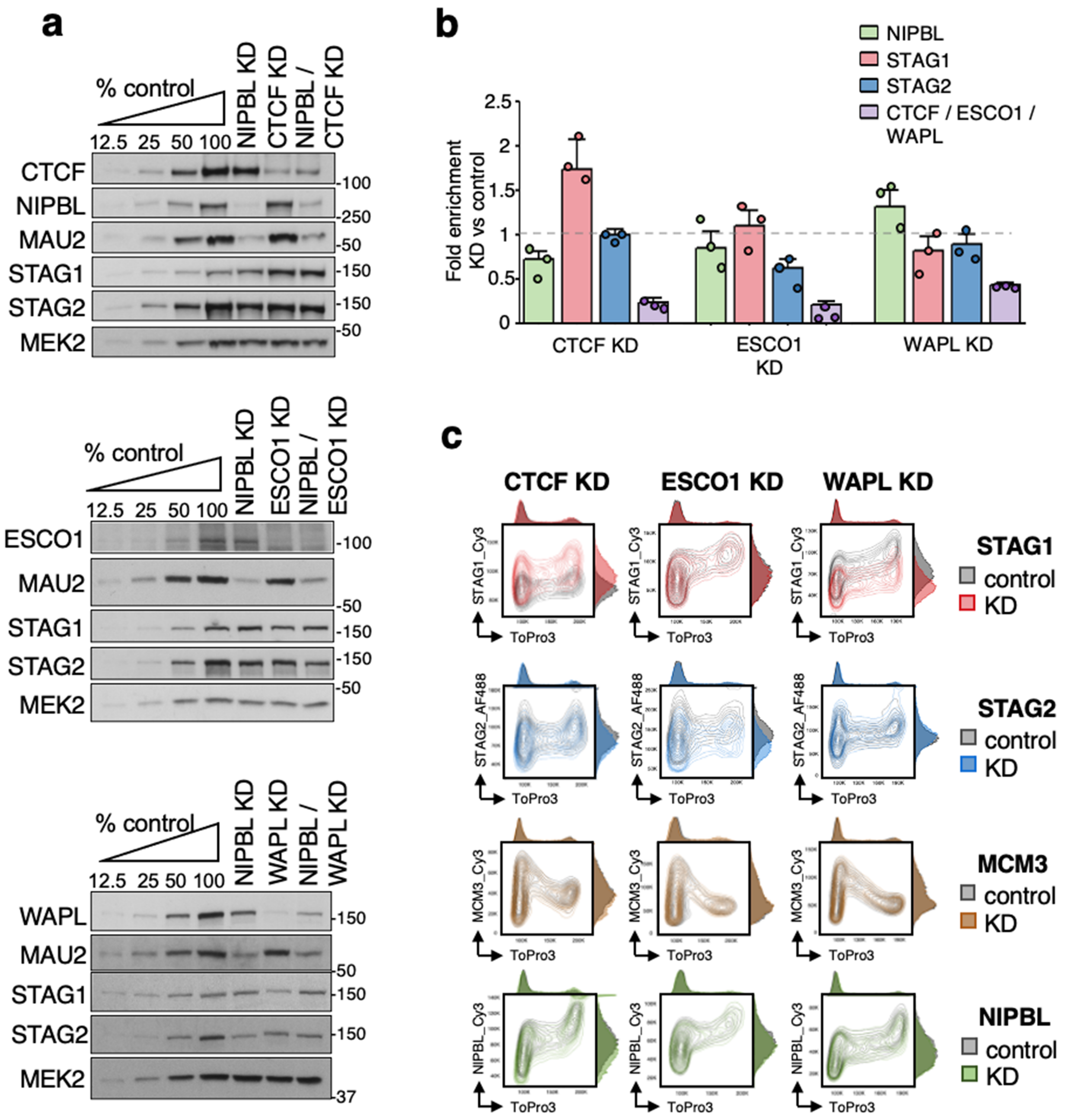

Supplementary Fig. 3 Effect of cohesin regulators on chromatin association of the two variants. a HeLa cells mock transfected (control) or transfected with siRNAs against CTCF, ESCO1 or WAPL (KD) were analyzed 72 h post-transfection by immunoblot. **b** RNA was extracted from the same cells for qRT-PCR analyses of the indicated genes. Results are represented as fold change of each KD condition compared to their respective controls and normalized to GAPDH. Data come from 3 experiments and are represented as mean values ±SEM. **c** Contour plots for chromatin bound proteins in control (grey) and KD cells (colored) in each condition were overlapped for comparison.

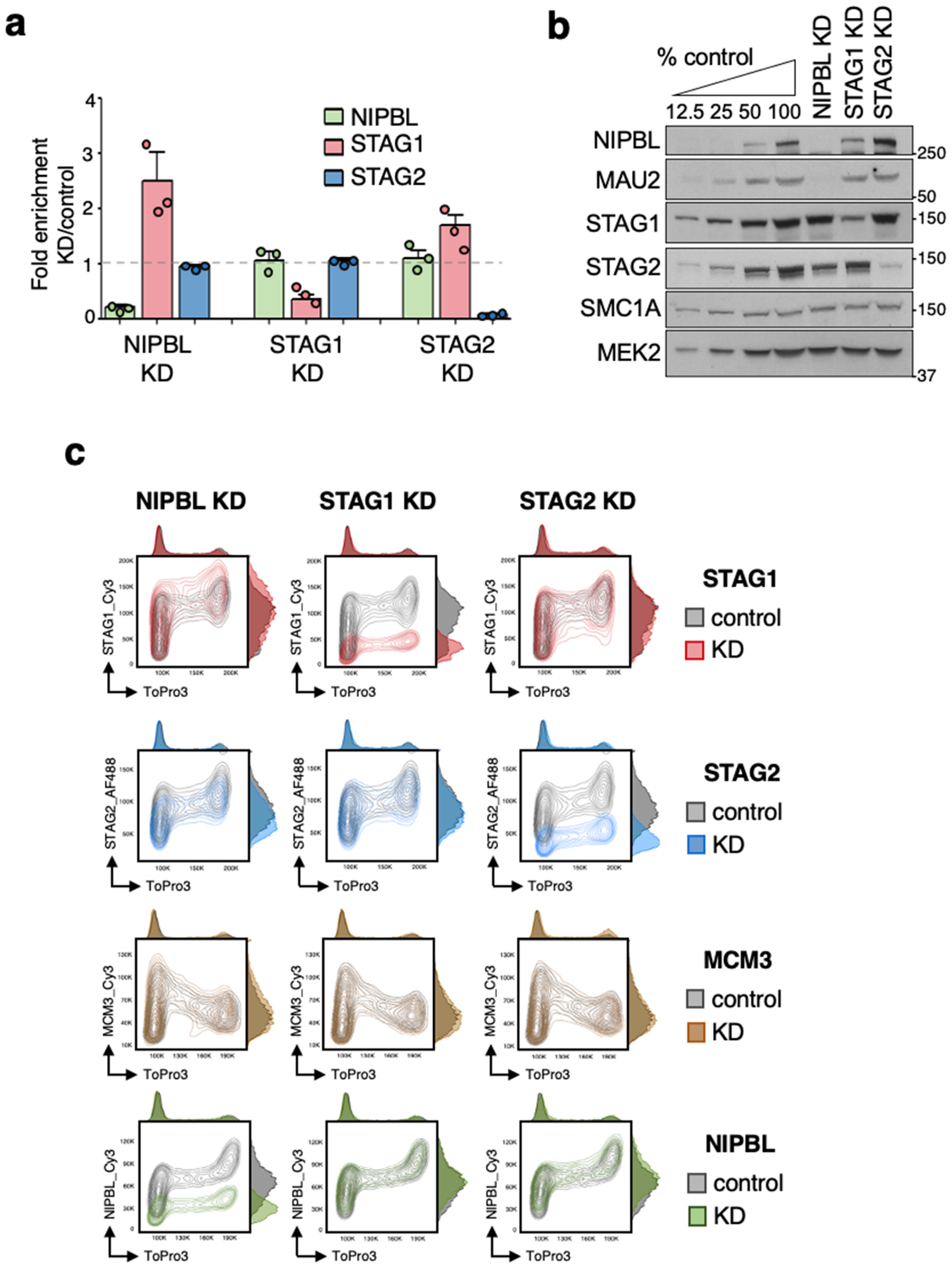

Supplementary Fig. 4 STAG1 behavior is not the same in NIPBL KD and STAG2 KD cells. a Quantification of mRNA levels of STAG1, STAG2 and NIPBL in the indicated KD cells expressed as fold change compared to their respective controls and normalized to GAPDH. Data from 3 experiments are represented as mean values ±SEM. **b** Immunoblot analyses of total cell extracts from control and KD HeLa cells. **c** Flow cytometry contour plots for the indicated chromatin-bound proteins in control cells (grey plots) and cells KD for NIPBL, STAG1 or STAG2 (colored) were overlapped for comparison. For b and c, a representative experiment out of the 3 performed is shown.

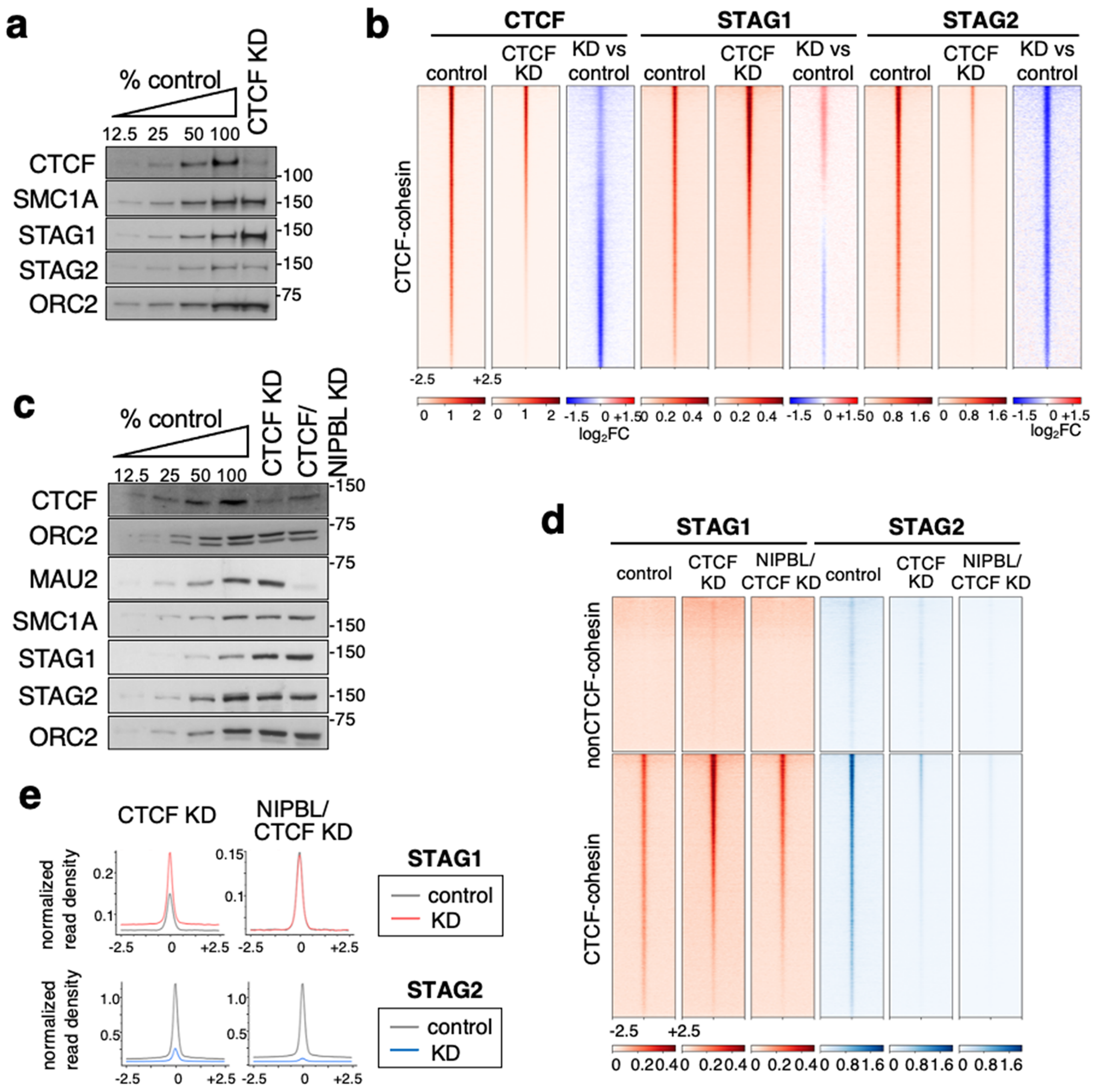

Supplementary Fig. 5 Cohesin-STAG1 persists at CTCF sites after reduction of CTCF and NIPBL levels. **a** Immunoblot analysis of CTCF KD cells used for ChIP-seq shown in Fig. 4c. Replicates are shown in c and Supplementary Fig. 3a. **b** Heatmap showing log2 fold change (log2FC) in CTCF KD versus control at CTCF-cohesin positions for CTCF, STAG1 and STAG2 ChIP signals. **c** Immunoblot analyses of cells used for ChIP-seq shown in d. **d** Heatmaps showing genome-wide distribution of STAG1 and STAG2 in MCF10A cells control, CTCF KD and double CTCF/NIPBL KD conditions. Reads from calibrated ChIP-seq are plotted in a 5-kb window centered in the summits of cohesin positions with and without CTCF. A single replicate for each condition is plotted. **e** Normalized read density plots for cohesin subunits ±2.5 kb of the summit in the different KD conditions.

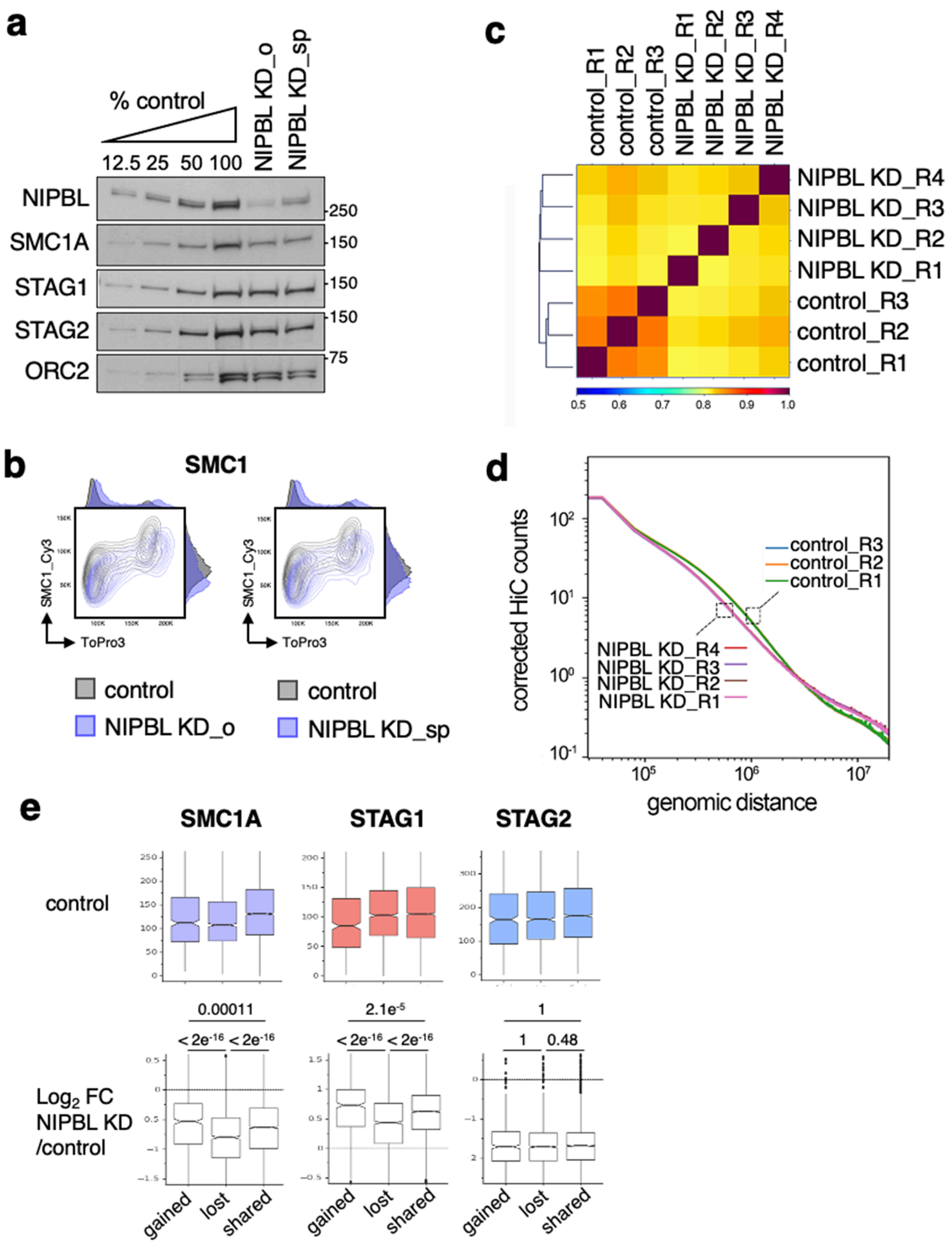

Supplementary Fig. 6 Correlation among Hi-C replicates and additional analyses. a Immunoblot analysis of the NIPBL KD cells used for in situ Hi-C. Cells were transfected with a single oligonucleotide (NIPBL KD_o) or with a smart pool of four oligonucleotides (NIPBL KD_sp). This is a single experiment. **b** Contour plots showing chromatin-bound SMC1A levels in NIPBL KD and control cells used in in situ Hi-C. **c** Hierarchical clustering of Hi-C data showing correlation among the replicates for the control (3) and NIPBL KD (4) conditions. **d** Contact probability as a function of genome distance in replicates of control and NIPBL KD cells. **e** Boxplots showing occupancy of the indicated proteins (SMC1A, STAG1, STAG2) in control cells at loop anchors for gained (406), lost (1029) and shared loops (2666) between control and NIPBL KD cells (colored plots) and the log2 fold change of this occupancy (uncolored plots). Boxes represent interquartile range (IQR); the midline represents the median; whiskers are 1.5 x IQR; and individual points are outliers. Statistical significance was calculated using a non-parametric Mann Whitney two-sided test with Holm's correction for multiple comparisons.

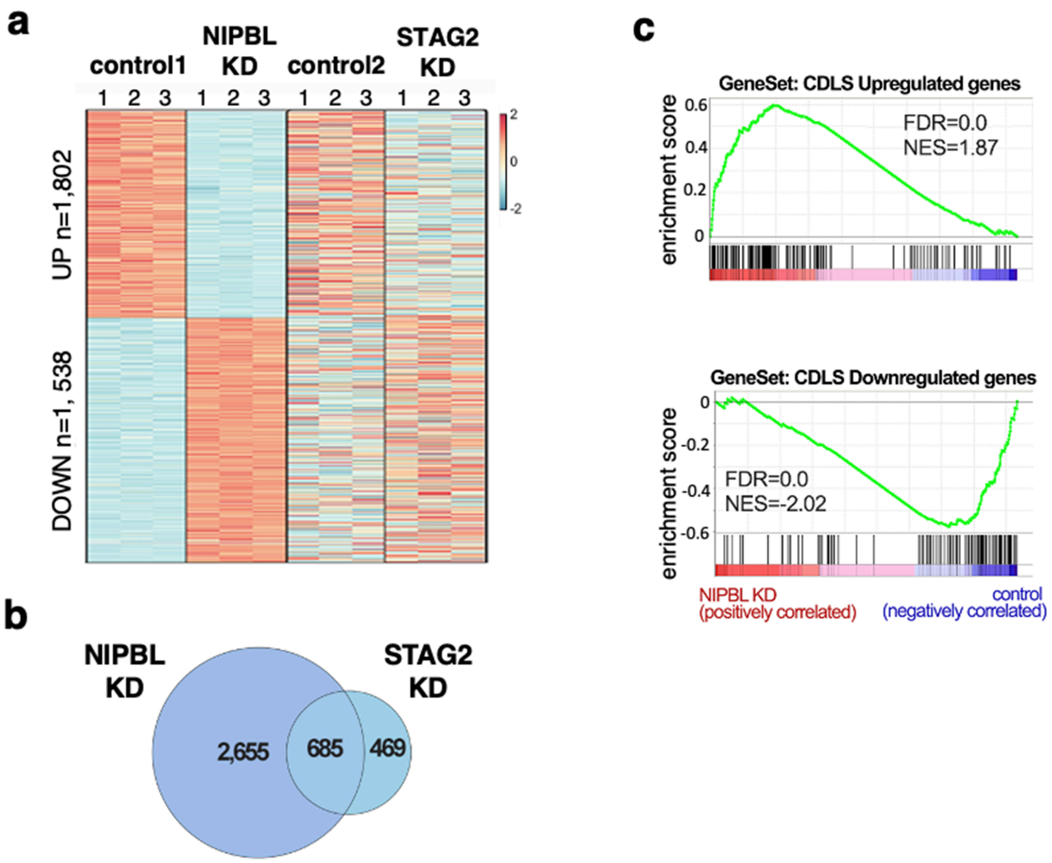

Supplementary Fig. 7 Gene deregulation in NIPBL KD cells resembling Cornelia de Lange Syndrome. a Heatmap of significant gene expression changes (FDR<0.05, |log2FC|>0.5) between MCF10A cells in control and NIPBL KD condition (3 replicates each) and comparison with the changes detected in STAG2 KD cells. **b.** Venn diagram showing Differentially Expressed Genes (DEGs) in the two KD conditions. See Supplementary Data 2 and 3 for gene lists. **c** GSEA was used to compare gene deregulation in NIPBL KD and STAG2 KD in MCF10A cells with that observed in lymphocyte cell lines from CdLS patients carrying mutations in NIPBL (see Supplementary Data 4 for genes in each geneset). Only NIPBL KD deregulated genes showed significant enrichment in gene sets encompassing CdLS upregulated (top) and downregulated (bottom) genes.

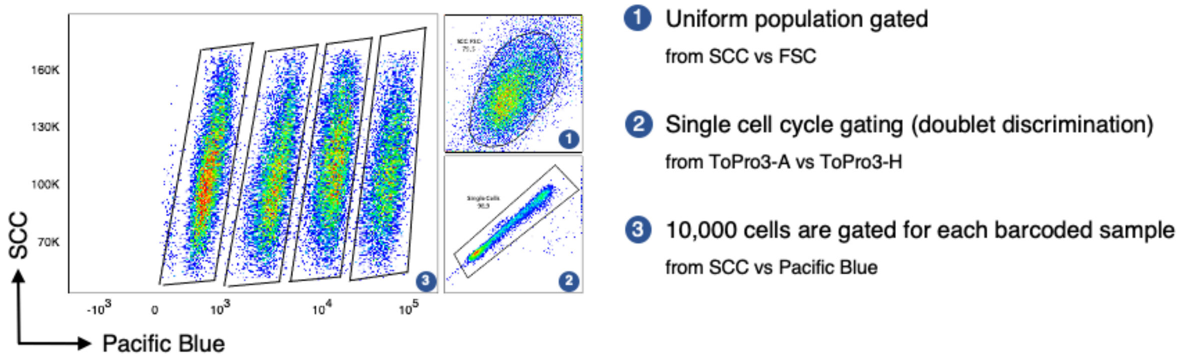

Supplementary Fig. 8. Gating strategy for flow cytometry analyses. The following steps were followed. First, the whole barcoded sample is gated by plotting forward scatter (FSC) versus side scatter (SSC), which measure cell size and granularity/complexity, respectively, in order to exclude cell debris and ensure a uniform population. Second, DNA content is plotted to gate single cells and avoid cell aggregates. Finally, the four barcoded populations stained with increasing concentrations of dye are separated by plotting SCC versus Pacific Blue. Each population is gated and 10,000 cells are exported for subsequent analysis.

Supplementary Table 1. Changes in cohesin subunit abundance on chromatin after NIPBL KD measured by immunofluorescence (related to Fig. 1c)
HeLa cells pre-extracted with detergent before fixation were stained with cohesin antibodies. Changes in staining intensity in NIPBL KD compared to control cells are expressed as percentage. Statistical significance was calculated with a non-parametric Mann Whitney two-sided test with confidence intervals of 99%

staining	condition	Mean intensity	SD	nº cells	% change	p value
**SMC1**	control	103.93	15.06	372	83%	<2E-16
	NIPBL KD	85.86	18.76	378		
**STAG1**	control	62.36	15.64	419	129%	<2E-16
	NIPBL KD	80.34	25.19	559		
**STAG2**	control	65.80	17.66	419	77%	< 2E-16
	NIPBL KD	51.10	12.04	559		

Supplementary Table 2. *STAG1* upregulation in NIPBL deficient cells

Cell*_condition	Log2FC	FDR	ref
LCLs_CdLS	1.16	0.02	[1]
MCF10A_siNIPBL	2.01	3.6E-200	this study
HAP1_*MAU2* KO	0.68	0.0008	[2]
P19_siNIPBL	0.52	7.8E-8	[3]
Hepatocytes_*Nipbl* KO	1.01	0.02	[4]
Embryonic brain_*Nipbl* +/-	0.21	0.005	[5]
MEFs_*Nipbl* +/-	0.53	0.009	[5]

*LCL, lymphoblastoid cell lines; MEF, mouse embryo fibroblasts

Supplementary Table 3. Differential peaks called in control and NIPBL KD cells (related to Figure 4a)

	FDR< 0.05			FDR <0.01		
**antibody_condition**	**peak number**	**common peaks**	**% peaks KD vs control**	**peak number**	**common peaks**	**% peaks KD vs control**
SMC1A_control	35,171	12,742	45.3%	30,305	9,100	34.9%
SMC1A_ NIPBL KD	15,945			10,574		
STAG1_control	26,355	22,524	110.9%	22,849	19,872	110.1%
STAG1_NIPBL KD	29,232			25,167		
STAG2_control	55,059	7,852	15.3%	46,577	5,936	13.4%
STAG2_NIPBL KD	8,413			6,247		

Supplemetary Table 4. Datasets used in this study

Type of data	Sample Name	Unique reads aligned to human (mill)	GEO accession	URL	Source
ChIP-seq	Input_Control1	30.8	GSE207116	https://www.ncbi.nlm.nih.gov/geo/query/acc.cgi?acc=GSE207116	this study
ChIP-seq	Input _NIPBL KD	33.6	GSE207116		this study
ChIP-seq	SMC1_Control1	53.2	GSE207116		this study
ChIP-seq	SMC1_NIPBL KD	23.6	GSE207116		this study
ChIP-seq	STAG1_Control1	25.6	GSE207116		this study
ChIP-seq	STAG1_NIPBL KD	24.8	GSE207116		this study
ChIP-seq	STAG2_Control1	34.8	GSE207116		this study
ChIP-seq	STAG2_NIPBL KD	25.0	GSE207116		this study
ChIP-seq	Input_Control2	42.6	GSE207116	https://www.ncbi.nlm.nih.gov/geo/query/acc.cgi?acc=GSE207116	this study
ChIP-seq	Input_CTCF KD	21.9	GSE207116		this study
ChIP-seq	Input_CTCF&NIPBL KD	26.5	GSE207116		this study
ChIP-seq	STAG1_Control2	24.9	GSE207116		this study
ChIP-seq	STAG1_CTCF KD	35.9	GSE207116		this study
ChIP-seq	STAG1_CTCF&NIPBL KD	24.9	GSE207116		this study
ChIP-seq	STAG2_Control2	10.2	GSE207116		this study
ChIP-seq	STAG2_CTCF KD	27.5	GSE207116		this study
ChIP-seq	STAG2_CTCF&NIPBL KD	34.9	GSE207116		this study
ChIP-seq	Input_Control3	31.9	GSE207116		this study
ChIP-seq	Input_CTCF KD2	33.3	GSE207116		this study
ChIP-seq	CTCF_Control	19.7	GSE207116		this study
ChIP-seq	CTCF_CTCF KD	28.5	GSE207116		this study
ChIP-seq	STAG1_Control3	26.7	GSE207116		this study
ChIP-seq	STAG1_CTCF KD2	26.6	GSE207116		this study
ChIP-seq	Input		GSM2599090	https://www.ncbi.nlm.nih.gov/geo/query/acc.cgi?acc=GSE98551	[6]
ChIP-seq	CTCF_R1		GSM2599084		[6]
ChIP-seq	CTCF_R2		GSM2599085		[6]
ChIP-seq	Input		GSM2718671	https://www.ncbi.nlm.nih.gov/geo/query/acc.cgi?acc=GSE101921	[7]
ChIP-seq	STAG1		GSM2718667		[7]
ChIP-seq	STAG2		GSM2718668		[7]
ChIP-seq	SMC1A		GSM2718669		[7]
ChIP-seq	Input_control		GSM2942299	https://www.ncbi.nlm.nih.gov/geo/query/acc.cgi?acc=GSE101921	[7]
ChIP-seq	STAG1_control R1,R2		GSM2942285, GSM2		[7]
ChIP-seq	STAG2_control R1,R2		GSM2942287, GSM2		[7]
ChIP-seq	Input_STAG2 KD		GSM2942297		[7]
ChIP-seq	STAG1_STAG2 KD R1,R2		GSM2942293,GSM2		[7]
ChIP-seq	STAG2_STAG2 KD R1,R2		GSM2942295,GSM2		[7]
Hi-C	control_R1	268.2	GSE207116	https://www.ncbi.nlm.nih.gov/geo/query/acc.cgi?acc=GSE207116	this study
Hi-C	control_R2	365.1	GSE207116		this study
Hi-C	control _R3	277.8	GSE207116		this study
Hi-C	NIPBL KD_R1 (smartpool_2)	291.3	GSE207116		this study
Hi-C	NIPBL KD_R2 (oligo_1)	260.1	GSE207116		this study
Hi-C	NIPBL KD_R3 (smartpool_1)	193.8	GSE207116		this study
Hi-C	NIPBL KD_R4 (oligo_2)	272.8	GSE207116		this study
Hi-C	control, R1,R2		GSM3110157, GSM3	https://www.ncbi.nlm.nih.gov/geo/query/acc.cgi?acc=GSE101921	[7]
Hi-C	STAG2 KD R1,R2		GSM3110161, GSM3		[7]
RNA-seq	Control (3 replicates)	25.4+31.9+31.8	GSE207116	https://www.ncbi.nlm.nih.gov/geo/query/acc.cgi?acc=GSE207116	this study
RNA-seq	NIPBL KD (3 replicates)	36.1+29.7+32,7	GSE207116		this study
RNA-seq	Control (3 replicates)		GSM2718676-8	https://www.ncbi.nlm.nih.gov/geo/query/acc.cgi?acc=GSE101921	[7]
RNA-seq	STAG2 KD (3 replicates)		GSM2718682-4		[7]
Espression profiling array	CdLS probands		GSE12408	https://www.ncbi.nlm.nih.gov/geo/query/acc.cgi?acc=GSE12408	[1]

Supplementary Table 5. In situ Hi-C statistics (QC)

Sample	Reads (mill pairs)	Mappable, unique, high quality (%)	HiC contacts	%trans contacts	% cis short (<20 kb)	% cis long (>20 kb)
control_R1	310.4	65	157,967,731	11.7	28.4	59.9
control_R2	388.8	72.8	253,688,297	10.7	28.8	60.5
control _R3	305.6	70.4	180,027,808	11.6	28.4	60.0
**Control_merge**	**1004.8**					
NIPBL KD_R1 (smartpool_2)	309.9	72.5	200,394,043	14.3	29.3	56.5
NIPBL KD_R2 (oligo_1)	277.0	73.2	179,769,165	13.7	29.1	57.1
NIPBL KD_R3 (smartpool_1)	206.8	71.9	134,240,661	14.3	28.4	57.3
NIPBL KD_R4 (oligo_2)	309.0	68.3	166,116,268	14.0	30.2	57.7
**NIPBL KD_merge**	**1102.7**					

Supplementary Table 6. Oligonucleotides

TYPE	NAME	SOURCE	SEQUENCE (or CAT#)
siRNA	hNIPBL custom	Dharmacon	5'-CUGAUAAACUAGAACGAAA-3'
siRNA	hNIPBL ON-TARGETplus SMARTpool	Dharmacon	L-012980-00
siRNA	hSTAG1 ON-TARGETplus SMARTpool	Dharmacon	L-010638-01
siRNA	hSTAG2 ON-TARGETplus SMARTpool	Dharmacon	L-021351-00
siRNA	hSMC1A ON-TARGETplus SMARTpool	Dharmacon	L-006833-00
siRNA	hCTCF ON-TARGETplus SMARTpool	Dharmacon	L-020165-00
siRNA	hESCO1 siGENOME SMARTpool	Dharmacon	M-023413-01
siRNA	hWAPL custom siRNA	Dharmacon	5'-CGGACUACCCUUAGCACAA-3'
DNA	gSTAG1 fw	IDT	5'- CACCGGATCGATTCAATCATTCTG -3'
DNA	gSTAG1 rev	IDT	5'- AAACCAGAATGATTGAATCGATCC -3'
DNA	gSTAG2 fw	IDT	5'- CACCGATTTCGACATACAAGCACCC -3'
DNA	gSTAG2 rev	IDT	5'- AAACGGGTGCTTGTATGTCGAAATC -3'
DNA	hNIPBL fw	IDT	5'-ACAGGCATGACAATAGGAGGGATTC-3'
DNA	hNIPBL rev	IDT	5'-ATCGCCCATCAGGTCTCTGC-3'
DNA	hSTAG1 fw	IDT	5'-CCTGGGAGTCTGACAAACCCG-3'
DNA	hSTAG1 rev	IDT	5'-TCCTCTTACCATGCCTGGACC-3'
DNA	hSTAG2 fw	IDT	5'-AGCTGGTGGTGATGATGACA-3'
DNA	hSTAG2 rev	IDT	5' CATAACAGGGGTGTGCAGTG-3'
DNA	hCTCF fw	IDT	5'-TCACCCTCCTGAGGAATCCAC-3'
DNA	hCTCF rev	IDT	5'-CGTAATCGCACATGGAACAC-3'
DNA	hESCO1 fw	IDT	5'-ACAGCATCTGCTTTTCCACA-3'
DNA	hESCO1 rev	IDT	5'-CCTGCCATCAGGGTATTCAG-3'
DNA	hWAPL fw	IDT	5'-AGCCTCTGCCTCACCAGAATG-3'
DNA	hWAPL rev	IDT	5'-TGTGCCTATGAGACCGTCCTG-3'
DNA	hGAPDH fw	IDT	5'-TGCACCACCAACTGCTTAGC-3'
DNA	hGAPDH	IDT	5'-GAGGGGCCATCCACAGTCTTC-3'

Supplementary Table 7. Antibodies

Target	Host	Source or Reference#	Immunogen	Application**/Dilution
NIPBL	Rabbit polyclonal*	Custom made, this study	recombinant fragment: mNIPBL aa2349-2667	FC: **4** μg/ml; WB: 5 μg/ml
MAU2	Rabbit monoclonal	Clone EPR14390, Abcam, ab183033	Recombinant fragment, undisclosed	WB: 1:1000
STAG1	Rat monoclonal	Custom made, clone SUSI 63B, ref [7]	recombinant fragment: mSTAG1 aa1-225	WB: 1:10
STAG1	Rabbit polyclonal*	Custom made, ref [8]	CEDDSGFGMPMF	FC: 4 μg/ml; WB: 2 μg/ml; IF: 1μg/ml
				ChIP: **50** μg/ 10e7 cells
STAG2	Rabbit polyclonal*	Custom made, ref [8]	CDPASIMDESVLGVSMF	ChIP: 50 μg/ 10e7 cells
STAG2	Mouse monoclonal	Clone J-12, SCBT, SC-81852	recombinant protein with epitope at C-term	FC: 2 μg/ml; WB: 0.4 μg/ml; IF: 1 μg/ ml
SMC1A	Rabbit polyclonal*	Custom made, ref [9]	CDLTKYPDANPNPNEQ	FC: 4 μg/ml; WB: 1 μg/ml; IF: 1μg/ ml
				ChIP: 50 μg/ 10e7 cells
CTCF	Rabbit polyclonal	Millipore 07-729 RRID:AB_441965	TNQPKQNQPTAIIQVED aa659-675 of hCTCF	ChIP: **15** μg/10e7 cells
CTCF	Rat monoclonal	Custom made, clone MARS159A/D1, this study	recombinant fragment: hCTCF aa574-727	WB: Undiluted supernatant
ESCO1	Mouse monoclonal	Custom made, ref [10], kind gift from K. Shirahige	CEEKLPVIRSEEEKVRFERQKA	WB: 1:200
WAPL	Rat monoclonal	Custom made, clone WAPI 432E/E9, ref [13]	recombinant fragment: hWAPL aa838-1190	WB: Undiluted supernatant
ORC2	Rabbit polyclonal	Custom made, ref [11], kind gift from J. Méndez	GST-hOrc2	WB: 1:1000
MCM3	Rabbit polyclonal	Custom made, ref [12], kind gift from J. Méndez	hMcm3, aa 674 -693	FC: 1:100; WB: 1:1000
MEK2	Mouse monoclonal	Clone 96/MEK2 (RUO), BD Bioscience AB_397631	recombinant fragment, Rat MEK2 1-110aa	WB: 1:1000
Cy3 AffiniPure	Rabbit polyclonal	Jackson ImmunoResearch: AB_2307443	IgG (H+L)	FC: 1:400
Alexa Fluor 488 AffiniPure	Mouse polyclonal	Jackson ImmunoResearch: AB_2340846	IgG (H+L)	FC: 1:400

* All custom made Rabbit polyclonal antibodies are affinity purified, except those for ORC2 and MCM3 [illegible]

**FC, Flow Cytometry; WB, Western Blot; IF, Immunofluorescence; ChIP, Chromatin Immunoprecipitation

Supplementary References

1. Liu, J. *et al.* Transcriptional dysregulation in NIPBL and cohesin mutant human cells. *PLoS Biol* **7**, e1000119 (2009).
2. Haarhuis, J. H. I. *et al.* The Cohesin Release Factor WAPL Restricts Chromatin Loop Extension. *Cell* **169**, 693–707 (2017).
3. Luna-Peláez, N. *et al.* The Cornelia de Lange Syndrome-associated factor NIPBL interacts with BRD4 ET domain for transcription control of a common set of genes. *Cell Death Dis.* **10**, 548 (2019).
4. Schwarzer, W. *et al.* Two independent modes of chromatin organization revealed by cohesin removal. *Nature* **551**, 51–56 (2017).
5. Kawauchi, S. *et al.* Multiple organ system defects and transcriptional dysregulation in the Nipbl(+/-) mouse, a model of Cornelia de Lange Syndrome. *PLoS Genet* **5**, e1000650 (2009).
6. Fritz, A. J. *et al.* Intranuclear and higher-order chromatin organization of the major histone gene cluster in breast cancer. *J. Cell. Physiol.* **233**, 1278–1290 (2018).
7. Kojic, A. *et al.* Distinct roles of cohesin-SA1 and cohesin-SA2 in 3D chromosome organization. *Nat. Struct. Mol. Biol.* **25**, 496–504 (2018).
8. Remeseiro, S. *et al.* Cohesin-SA1 deficiency drives aneuploidy and tumourigenesis in mice due to impaired replication of telomeres. *EMBO J* **31**, 2076–2089 (2012).
9. Remeseiro, S., Cuadrado, A., Gómez-López, G., Pisano, D. G. & Losada, A. A unique role of cohesin-SA1 in gene regulation and development. *EMBO J* **31**, 2090–2102 (2012).
10. Minamino, M. *et al.* Esco1 Acetylates Cohesin via a Mechanism Different from That of Esco2. *Curr Biol* **25**, 1694–1706 (2015).
11. Gavin, K. A., Hidaka, M. & Stillman, B. Conserved initiator proteins in eukaryotes. *Science (80-.).* **270**, 1667–1671 (1995).
12. Mendez, J. & Stillman, B. Chromatin association of human origin recognition complex, cdc6, and minichromosome maintenance proteins during the cell cycle: assembly of prereplication complexes in late mitosis. *Mol Cell Biol* **20**, 8602–8612 (2000).
13. Morales, C. *et al.* PDS5 proteins are required for proper cohesin dynamics and participate in replication fork protection. *J. Biol. Chem.* **2895**, 146–157 (2020).
