# Supplementary material for: Different NIPBL requirements of cohesin-STAG1 and cohesin-STAG2
Source: Nat Commun. 2023 Mar 10;14:1326. doi: 10.1038/s41467-023-36900-7 (PMC10006224; doi:10.1038/s41467-023-36900-7)
Supplement: Supplementary file 3 — Description of Additional Supplementary Files [file 41467_2023_36900_MOESM3_ESM.pdf]

Description of Additional Supplementary Files

File Name: **Supplementary Data 1**.
Description: **Called loops shared, gained and lost in NIPBL KD condition.** Loops were called at 10-kb resolution in each replicate and only those loops that were called at least twice among all the replicates were considered for subsequent analyses. We next defined “gained” loops as those called only in NIPBL KD replicates and “lost” loops as those called only in wild type replicates. The rest of the loops were considered “shared” loops.

File Name: **Supplementary Data 2.**
Description: **Differentially Expressed Genes (DEGs) in NIPBL KD cells.** DEGs were obtained with DEseq2 package with three replicates per condition using $FDR<0.05$ and $|log2FC|>0.5$.

File Name: **Supplementary Data 3.**
Description: **Differentially Expressed Genes (DEGs) in STAG2 KD cells.** Data from ref. 8 were reanalyzed. DEGs were obtained with DEseq2 package with three replicates per condition using $FDR<0.05$ and $|log2FC|>0.5$.

File Name: **Supplementary Data 4.**
Description: **CdLS gene sets.** List of genes composing the gene set “CdLS upregulated genes” and “CdLS downregulated genes” corresponding to genes differentially expressed in lymphoid cell lines from CdLS patients and healthy controls with $FDR<0.01$ from ref. [1]
