# Supplementary material for: Different NIPBL requirements of cohesin-STAG1 and cohesin-STAG2
Source: Nat Commun. 2023 Mar 10;14:1326. doi: 10.1038/s41467-023-36900-7 (PMC10006224; doi:10.1038/s41467-023-36900-7)
Supplement: Supplementary file 8 — Reporting Summary [file 41467_2023_36900_MOESM8_ESM.pdf]

nature portfolio

Corresponding author(s): Ana Losada

Last updated by author(s): Feb 2, 2023

Reporting Summary

Nature Portfolio wishes to improve the reproducibility of the work that we publish. This form provides structure for consistency and transparency in reporting. For further information on Nature Portfolio policies, see our Editorial Policies and the Editorial Policy Checklist.

Statistics

For all statistical analyses, confirm that the following items are present in the figure legend, table legend, main text, or Methods section.

n/a	Confirmed	
☐	☒	The exact sample size (*n*) for each experimental group/condition, given as a discrete number and unit of measurement
☐	☒	A statement on whether measurements were taken from distinct samples or whether the same sample was measured repeatedly
☐	☒	The statistical test(s) used AND whether they are one- or two-sided<br>*Only common tests should be described solely by name; describe more complex techniques in the Methods section.*
☒	☐	A description of all covariates tested
☒	☐	A description of any assumptions or corrections, such as tests of normality and adjustment for multiple comparisons
☐	☒	A full description of the statistical parameters including central tendency (e.g. means) or other basic estimates (e.g. regression coefficient) AND variation (e.g. standard deviation) or associated estimates of uncertainty (e.g. confidence intervals)
☐	☒	For null hypothesis testing, the test statistic (e.g. *F*, *t*, *r*) with confidence intervals, effect sizes, degrees of freedom and *P* value noted<br>*Give P values as exact values whenever suitable.*
☒	☐	For Bayesian analysis, information on the choice of priors and Markov chain Monte Carlo settings
☒	☐	For hierarchical and complex designs, identification of the appropriate level for tests and full reporting of outcomes
☒	☐	Estimates of effect sizes (e.g. Cohen's *d*, Pearson's *r*), indicating how they were calculated

Our web collection on statistics for biologists contains articles on many of the points above.

Software and code

Policy information about availability of computer code

Data collection	FACS data was obtained on BD LSRII Fortessa flow cytometer using BD FACSDiva software; Fluorescent images were acquired in a TCS-SP5 (AOBS) Confocal microscope (Leica Microsystems) with LAS AF v2.6 acquisition software; NGS libraries were sequenced on an Illumina NextSeq550; qRT-PCR was performed on ABI Prism® 7900HT instrument (Applied Biosystems®);
Data analysis	FlowJo v10 software; Definiens Developer XD v2.5 software (Definiens); Bowtie2 (version 2.4.2) ; MACS2 (version 2.2.7.1); GATK4 (version 4.1.9.0); deepTools 3.5.0; HiCExplorer; coolpup.py, FastQC; Fastqscreen; Nextpresso; GSEA_4.2.3; R; Python

For manuscripts utilizing custom algorithms or software that are central to the research but not yet described in published literature, software must be made available to editors and reviewers. We strongly encourage code deposition in a community repository (e.g. GitHub). See the Nature Portfolio guidelines for submitting code & software for further information.

Data

Policy information about availability of data

All manuscripts must include a data availability statement. This statement should provide the following information, where applicable:

- Accession codes, unique identifiers, or web links for publicly available datasets
- A description of any restrictions on data availability
- For clinical datasets or third party data, please ensure that the statement adheres to our policy

NGS data (ChIP-seq, Hi-C and RNA-seq) has been deposited in GEO, accession number GSE207116 (https://www.ncbi.nlm.nih.gov/geo/query/acc.cgi?

acc=GSE207116). Additional datasets used are listed (with links) in Supplementary Table 4 and are also mentioned in the Data availability statement ("Additional datasets used include GSE101921 for ChIP-seq, RNA-seq and Hi-C data in STAG2 KD MCF10A cells (https://www.ncbi.nlm.nih.gov/geo/query/acc.cgi?acc=GSE101921), GSE98551 for CTCF distribution in MCF10A cells (https://www.ncbi.nlm.nih.gov/geo/query/acc.cgi?acc=GSE98551) and GSE12408 for expression profiling arrays of CdLS probands (https://www.ncbi.nlm.nih.gov/geo/query/acc.cgi?acc=GSE12408).

Human research participants

Policy information about studies involving human research participants and Sex and Gender in Research.

Reporting on sex and gender	N/A
Population characteristics	N/A
Recruitment	N/A
Ethics oversight	N/A

Note that full information on the approval of the study protocol must also be provided in the manuscript.

Field-specific reporting

Please select the one below that is the best fit for your research. If you are not sure, read the appropriate sections before making your selection.

☒ Life sciences ☐ Behavioural & social sciences ☐ Ecological, evolutionary & environmental sciences

For a reference copy of the document with all sections, see nature.com/documents/nr-reporting-summary-flat.pdf

Life sciences study design

All studies must disclose on these points even when the disclosure is negative.

Sample size	For Figure 1c, sample size was chosen based on similar experiments from other authors (e.g., Haarhuis et al 2017 Cell, http://dx.doi.org/10.1016/j.cell.2017.04.013, Figure 3F, "Quantitative immunofluorescence of chromatin-bound cohesin (...) n is at least 75 cells per sample"). We examined at least 372 cells per condition.
Data exclusions	none
Replication	Flow cytometry experiments comparing NIPBL KD and control cells were performed at least 3 times and in several cell lines. For other KD conditions, at least 3 times in HeLa. In some cases in which KD was not efficient, results were less clear, but with good KD efficienciies, results were robust. ChIP-seq in control and NIPBL KD was performed in single replicates with 3 different cohesin antibodies (SMC1, STAG1, STAG2); a second experiment was performed in control and CTCF KD for CTCF, STAG1 and STAG2 (Fig 4b) and a third one in control, CTCF KD ad double CTCF/NIPBL KD for STAG1 and STAG2 (Supplementary Fig 5e); for Hi-C, 3 replicates for control and 4 replicates for NIPBL KD (2x2 different s RNA oligos) were analyzed; for RNA-seq, triplicates of each condition were analyzed.
Randomization	no
Blinding	no

Reporting for specific materials, systems and methods

We require information from authors about some types of materials, experimental systems and methods used in many studies. Here, indicate whether each material, system or method listed is relevant to your study. If you are not sure if a list item applies to your research, read the appropriate section before selecting a response.

Materials & experimental systems

n/a	Involved in the study	
☐	☒	Antibodies
☐	☒	Eukaryotic cell lines
☒	☐	Palaeontology and archaeology
☒	☐	Animals and other organisms
☒	☐	Clinical data
☒	☐	Dual use research of concern

Methods

n/a	Involved in the study	
☐	☒	ChIP-seq
☐	☒	Flow cytometry
☒	☐	MRI-based neuroimaging

Antibodies

Antibodies used	Information of antibodies including source, publication, and indication for use in the different application is presented in Supplementary Table 7. Custom made: NIPBL, STAG1, STAG2, SMC1A, CTCF and WAPL (our group), ESCO1 (Shirahige lab),ORC2 and MCM3 (Méndez lab); commercial: MAU2 (abcam ab183033), STAG2 (SC-81852, SCBT), MEK2 (AB_397631,BD Bioscience).
Validation	Both custom made and commercial antibodies have been previously validated and used in publications with the exception of new custom made antibodies made for NIPBL, WAPL and CTCF. These have been used and validated for Western blot (e.g., Supplementary Fig. 3) and NIPBL also for Flow cytometry (e.g., Supplementary Fig. 2) using KD experiments reported in the manuscript. Antibodies for SMC1A, STAG1 and STAG2 have been previously used and validated for ChIP in human cells (Kojic 2018, DOI: 10.1038/s41594-018-0070-4, which include ChIP after STAG1 KD and STAG2 KD) and are validated here also for flow cytometry (e.g., Fig. 3, FC experiments in human A673 KO cells). ESCO1 antibody is validated for western blot in Minamino 2015 (DOI: 10.1016/j.cub.2015.05.017); ORC2 and MCM3 in Méndez and Stillman 2000 (DOI: 10.1128/MCB.20.22.8602-8612.2000). Commercial CTCF is used for ChIP in several studies (e.g. Pugacheva 2020 DOI: 10.1073/pnas.1911708117). MAU2 antibody should have been validated by the company (abcam) but we have also validated by western blot after MAU2 KD (not shown in manuscript) and after NIPBL KD (shown in most western blots in the paper).

Eukaryotic cell lines

Policy information about cell lines and Sex and Gender in Research

Cell line source(s)	HeLa cells (provided by G. Roncador, Monoclonal Antibodies Unit at CNIO, Madrid); MCF10A cells (provided by Dr. Quintela, CNIO, Madrid); A673 cells (provided by Dr. E. de Alava, IBIS, Sevilla). All originally purchased in ATCC.
Authentication	MCF10A was authenticated by karyotype analysis (CytogenDOI: 10.1016/j.cub.2015.05.017 etics Unit at CNIO); A673 and HeLa cell lines were authenticated byshort tandem repeat (STR) profiling (Genomics Unit, CNIO).
Mycoplasma contamination	All cells tested negative for mycoplasm with the GEN-PROBE MTC-NI rapid detection system.
Commonly misidentified lines (See ICLAC register)	None

ChIP-seq

Data deposition

☒ Confirm that both raw and final processed data have been deposited in a public database such as GEO.

☒ Confirm that you have deposited or provided access to graph files (e.g. BED files) for the called peaks.

Data access links *May remain private before publication.*	accession number GSE207116; https://www.ncbi.nlm.nih.gov/geo/query/acc.cgi?acc=GSE207116 token: sfszqwiuplklroj.
Files in database submission	GSM6278651 MCF10A_Control_R1_RNA-seq<br>GSM6278652 MCF10A_Control_R2_RNA-seq<br>GSM6278653 MCF10A_Control_R3_RNA-seq<br>GSM6278654 MCF10A_NIPBL KD_R1_RNA-seq<br>GSM6278655 MCF10A_NIPBL KD_R2_RNA-seq<br>GSM6278656 MCF10A_NIPBL KD_R3_RNA-seq<br>GSM6278699 MCF10A_Input_Control1_ChIP-seq<br>GSM6278700 MCF10A_Input _NIPBL KD_ChIP-seq<br>GSM6278701 MCF10A_SMC1_Control1_ChIP-seq<br>GSM6278702 MCF10A_SMC1_NIPBL KD_ChIP-seq<br>GSM6278703 MCF10A_STAG1_Control1_ChIP-seq<br>GSM6278704 MCF10A_STAG1_NIPBL KD_ChIP-seq<br>GSM6278705 MCF10A_STAG2_Control1_ChIP-seq<br>GSM6278706 MCF10A_STAG2_NIPBL KD_ChIP-seq<br>GSM6278707 MCF10A_Input_Control2_ChIP-seq<br>GSM6278708 MCF10A_Input_CTCF KD_ChIP-seq<br>GSM6278709 MCF10A_Input_CTCF-NIPBL KD_ChIP-seq<br>GSM6278710 MCF10A_STAG1_Control2_ChIP-seq<br>GSM6278711 MCF10A_STAG1_CTCF KD_ChIP-seq<br>GSM6278712 MCF10A_STAG1_CTCF-NIPBL KD<br>GSM6278713 MCF10A_STAG2_Control2_ChIP-seq<br>GSM6278714 MCF10A_STAG2_CTCF KD_ChIP-seq<br>GSM6278715 MCF10A_STAG2_CTCF-NIPBL KD_ChIP-seq<br>GSM6278716 MCF10A_Control_R1_HiC<br>GSM6278717 MCF10A_Control_R2_HiC<br>GSM6278718 MCF10A_Control_R3_HiC<br>GSM6278719 MCF10A_NIPBL KD_R1_HiC<br>GSM6278720 MCF10A_NIPBL KD_R2_HiC<br>GSM6278721 MCF10A_NIPBL KD_R3_HiC<br>GSM6278722 MCF10A_NIPBL KD_R4_HiC

GSM6675532 MCF10A_Input_Control3_ChIP-seq
GSM6675533 MCF10A_Input_CTCF-KD2_ChIP-seq
GSM6675534 MCF10A_STAG1_Control3_ChIP-seq
GSM6675535 MCF10A_STAG1_CTCF-KD2_ChIP-seq
GSM6675536 MCF10A_CTCF_Control3_ChIP-seq
GSM6675537 MCF10A_CTCF_CTCF-KD2_ChIP-seq

Genome browser session (e.g. UCSC)

http://genome-euro.ucsc.edu/s/Dinamica%20cromosomica/Reviewer_session_Alonso%20Gil

Methodology

Replicates

For RNA-seq, triplicates of each condition were analyzed; ChIP-seq was performed in single replicates with 3 different cohesin antibodies (SMC1, STAG1, STAG2); for Hi-C, 3 replicates for control and 4 replicates for NIPBL KD (2x2 different siRNA oligos) were analyzed.

Sequencing depth

Dataset, millions sequenced, millions uniquely aligned (single end)
MCF10A_Control_R1_RNA-seq, 33.6, 27.9
MCF10A_Control_R2_RNA-seq, 26.8, 22.5
MCF10A_Control_R3_RNA-seq, 33.4, 28.1
MCF10A_NIPBL KD_R1_RNA-seq, 31.2, 26.3
MCF10A_NIPBL KD_R2_RNA-seq, 37.9, 31.7
MCF10A_NIPBL KD_R3_RNA-seq, 34.4, 28.6
MCF10A_Input_Control1_ChIP-seq, 38.6, 34.1
MCF10A_Input _NIPBL KD_ChIP-seq, 42.4, 37.3
MCF10A_SMC1_Control1_ChIP-seq, 53.5+43, 37.1+30
MCF10A_SMC1_NIPBL KD_ChIP-seq, 33, 27.2
MCF10A_STAG1_Control1_ChIP-seq, 36.6, 29.2
MCF10A_STAG1_NIPBL KD_ChIP-seq, 35.6, 28
MCF10A_STAG2_Control1_ChIP-seq, 34.9+67.3, 28.8+26.5
MCF10A_STAG2_NIPBL KD_ChIP-seq, 34.9, 28.8
MCF10A_Input_Control2_ChIP-seq, 53.8, 42.6
MCF10A_Input_CTCF KD_ChIP-seq, 28, 21.9
MCF10A_Input_CTCF-NIPBL KD_ChIP-seq, 35.1, 26.5
MCF10A_STAG1_Control2_ChIP-seq, 29.1, 24.9
MCF10A_STAG1_CTCF KD_ChIP-seq, 41.7, 35.9
MCF10A_STAG1_CTCF-NIPBL KD, 29.4, 24.9
MCF10A_STAG2_Control2_ChIP-seq, 18.3, 10.2
MCF10A_STAG2_CTCF KD_ChIP-seq, 34.1, 27.5
MCF10A_STAG2_CTCF-NIPBL KD_ChIP-seq, 42.7, 34.9
MCF10A_Input_Control3_ChIP-seq, 35.1, 31.9
MCF10A_Input_CTCF-KD2_ChIP-seq, 36.8, 33.3
MCF10A_STAG1_Control3_ChIP-seq, 30.0, 26.7
MCF10A_STAG1_CTCF-KD2_ChIP-seq, 29.8, 26.6
MCF10A_CTCF_Control3_ChIP-seq 24.2, 19.7
MCF10A_CTCF_CTCF-KD2_ChIP-seq, 33.4, 28.5
Dataset, millions sequenced, millions uniquely aligned (paired-end)
MCF10A_Control_R1_HiC, 310.4, 158
MCF10A_Control_R2_HiC, 388.8, 254.89
MCF10A_Control_R3_HiC, 305.6, 178
MCF10A_NIPBL KD_R1_HiC, 309.9, 200.5
MCF10A_NIPBL KD_R2_HiC, 277, 179.8
MCF10A_NIPBL KD_R3_HiC, 206.8, 134.2
MCF10A_NIPBL KD_R4_HiC, 309, 166.2

Antibodies

Antibodies for SMC1, STAG1 and STAG2 are custom made and validated in a previous publication Kojic et al 2018, NSMB 25(6):496-504.doi: 10.1038/s41594-018-0070-4.

Peak calling parameters

Peak calling was carried out using MACS2 (version 2.2.7.1) after setting the q value (FDR) to 0.05 and using the '–extsize' argument with the values obtained in the 'macs2 predictd' step

Data quality

FastQC and Fastqscreen were used to measure quality of the reads

Software

Bowtie2 (version 2.4.2) ; MACS2 (version 2.2.7.1); deepTools 3.5.0; HiCExplorer; FastQC; Fastqscreen; Nextpresso; GSEA_4.2.3; R; Python

Flow Cytometry

Plots

Confirm that:

☒ The axis labels state the marker and fluorochrome used (e.g. CD4-FITC).

☒ The axis scales are clearly visible. Include numbers along axes only for bottom left plot of group (a 'group' is an analysis of identical markers).

☒ All plots are contour plots with outliers or pseudocolor plots.

☒ A numerical value for number of cells or percentage (with statistics) is provided.

Methodology

Sample preparation	HeLa and A673 cells were cultured in DMEM (BE12-604F/U1, Lonza) supplemented with 10% FBS and 1% penicillin-streptomycin. MCF10A cells were cultured in DMEM/F12 (#31330038, ThermoFisher) supplemented with 20 ng/ml EGF, 0.5 mg/ml hydrocortisone, 100 ng/ml cholera toxin, 10 mg/ml insulin and 5% horse serum. All cell lines were grown at 37°C under 90% humidity and 5% CO_2. To analyze chromatin bound proteins, cells were treated for 5 min with a low salt extraction buffer (0.1% Igepal CA-630, 10 mM NaCl, 5 mM $MgCl_2$, 0.1 mM PMSF, 10 mM Potassium Phosphate buffer pH 7.4) and fixed in 1% PFA final concentration. To evaluate the strength of chromatin association, cells were incubated for 5 minutes with salt extraction buffer containing 100 mM NaCl after the 5 min in low salt extraction buffer and before fixation. To analyze total proteins, unextracted cells were fixed in ice-cold 70% ethanol for 2 h. To eliminate antibody staining variation among samples from different conditions, a barcoding strategy was used. Four different samples were stained with increasing dilutions of Pacific Blue (Invitrogen) for 30 min in the dark at room temperature (RT) and then mixed into one tube. Then, each barcoded sample was blocked in flow buffer (0.1% Igepal CA-630, 6.5 mM Na_2HPO_4, 1.5 mM KH_2PO_4, 2.7 mM KCl, 137 mM NaCl, 0.5 EDTA pH 7.5, 4% non-fat milk) for 5 min and consecutively incubated with primary and secondary antibodies, also diluted in flow buffer, for 1 hour each. Finally, DNA staining was performed over night with 125 nM ToPRO3-iodide 642/661 in PBS.
Instrument	BD LSRII Fortessa
Software	BD FACSDiva software
Cell population abundance	Homogeneous cell cultures (HeLa, MCF10A or A673) were used in all experiments
Gating strategy	At least 10,000 cells were recorded for each population in a barcoded sample.

☒ Tick this box to confirm that a figure exemplifying the gating strategy is provided in the Supplementary Information.
